# Computed Tomography Angiography‐Assisted Ovarian Vein Sampling for Diagnosing an Androgen‐Producing Leydig Cell Tumor: A Case Report

**DOI:** 10.1155/crie/1283908

**Published:** 2026-01-20

**Authors:** Ken Tomotsune, Daisuke Saito, Satoshi Saitou, Yusuke Seki, Souta Hayashi, Yumiko Yamashita, Maiko Takata, Kentarou Ohara, Yohei Masugi, Yutaka Saito, Koki Kato, Tomotoshi Hosokawa

**Affiliations:** ^1^ Department of Diabetes and Endocrinology, Saiseikai Utsunomiya Hospital, 911-1 Takebayashimachi, Utsunomiya, 321-0974, Tochigi, Japan, saimiya.com; ^2^ Department of Obstetrics and Gynecology, Takata Clinic, 3039-2 Tomatsuricho, Utsunomiya, 320-0053, Tochigi, Japan; ^3^ Department of Pathology, Saiseikai Utsunomiya Hospital, 911-1 Takebayashimachi, Utsunomiya, 321-0974, Tochigi, Japan, saimiya.com; ^4^ Department of Pathology, Tokai University School of Medicine, 143 Shimokasuya, Isehara, 259-1193, Kanagawa, Japan, u-tokai.ac.jp; ^5^ Department of Radiology, Saiseikai Utsunomiya Hospital, 911-1 Takebayashimachi, Utsunomiya, 321-0974, Tochigi, Japan, saimiya.com; ^6^ Department of Obstetrics and Gynecology, Saiseikai Utsunomiya Hospital, 911-1 Takebayashimachi, Utsunomiya, 321-0974, Tochigi, Japan, saimiya.com

**Keywords:** computed tomography angiography, fertility-sparing surgery, hyperandrogenism, Leydig cell tumor, ovarian vein sampling

## Abstract

**Background:**

Ovarian Leydig cell tumors, rare androgen‐producing neoplasms, may present with rapidly progressive virilization. Preoperative localization is frequently challenging, particularly when bilateral ovarian abnormalities coexist or radiologic findings are equivocal. To avoid unnecessary bilateral oophorectomy and preserve fertility in reproductive‐aged females, accurate localization is crucial.

**Case Presentation:**

We describe the case of a 42‐year‐old reproductive‐aged female who presented with hirsutism, voice deepening, amenorrhea, and biochemical evidence of severe hyperandrogenism. Imaging revealed a solid nodule‐like lesion in the right ovary and a large cystic lesion in the left ovary, making the true origin of androgen excess uncertain. To achieve definitive localization, preprocedural computed tomography (CT) angiography‐guided ovarian vein sampling (OVS) was performed, which delineated the venous anatomy and facilitated successful right ovarian vein catheterization. Hormonal analysis revealed markedly elevated testosterone levels in the right ovarian vein, confirming unilateral secretion. Laparoscopic right adnexectomy and left ovarian cystectomy were performed. Pathological analysis confirmed a right ovarian Leydig cell tumor and a benign left ovarian cyst. Postoperatively, serum testosterone levels normalized, menstruation resumed, and virilization features gradually improved over 1 year.

**Conclusion:**

This case underscores the utility of CT angiography‐assisted OVS in localizing androgen‐producing ovarian tumors when conventional imaging is inconclusive. Functional confirmation of laterality enabled fertility‐sparing surgery (FSS) in a reproductive‐aged patient and led to optimal clinical outcomes. Adapting adrenal vein sampling (AVS) methodology to OVS represents a valuable diagnostic approach in selected cases of severe hyperandrogenism.

## 1. Introduction

Hyperandrogenism in females can arise from adrenal or ovarian sources, and differentiating between these etiologies is frequently challenging. Although biochemical assessment typically offers evidence of androgen excess, precisely localizing the source may be elusive when lesions are small, bilateral abnormalities are present, or radiologic findings are equivocal. Among ovarian androgen‐secreting tumors, Leydig cell tumors are particularly rare, accounting for <0.1% of all ovarian neoplasms, and typically present with rapid virilization, including hirsutism, voice deepening, and amenorrhea [1–5].

Accurate preoperative localization is essential for definitive treatment and avoiding unnecessary bilateral oophorectomy in young females in whom fertility preservation is of paramount significance. However, conventional imaging often fails to identify these small tumors, leading to inappropriate surgical decisions, as observed in previously reported cases [6–8]. Although advanced modalities, including positron emission tomography/computed tomography (CT), have been reported to localize ovarian Leydig cell tumors in selected cases [9], their use remains limited in routine clinical practice.

Selective venous sampling (SVS) has become a valuable diagnostic tool in endocrine practice when noninvasive modalities are inconclusive. Adrenal vein sampling (AVS) is a well‐established procedure for primary aldosteronism, and its application has expanded to other endocrine disorders, including hyperandrogenism [10–14]. Recent advances, including preprocedural CT angiography and better catheterization techniques, have increased the success rates of AVS [15, 16] and may enhance the performance of ovarian vein sampling (OVS), which is conventionally limited by technical challenges, particularly in accessing the right ovarian vein [6, 12].

We herein report the case of a 42‐year‐old woman with severe hyperandrogenism in whom bilateral ovarian abnormalities were identified, and functional localization using CT angiography‐assisted OVS facilitated the accurate lateralization of androgen secretion, which directly guided a fertility‐sparing surgery (FSS).

## 2. Case Presentation

A 42‐year‐old unmarried woman was referred to our hospital with the chief complaints of male pattern baldness, hypertrichosis with beard growth, voice deepening, and secondary amenorrhea. The patient had been experiencing male pattern baldness and voice deepening for 6 months before amenorrhea onset and hypertrichosis, including beard growth, for 3 months. She recently noticed an increase in facial acne. Consequently, she sought consultation at an external gynecologic clinic.

Her blood test results were as follows: significantly elevated free testosterone level at 11.0 (normal range, 0.3–1.8 pg/mL); estradiol level at the lower end of the normal range (50 pg/mL; 28.8–197 pg/mL in the follicular phase); and low‐normal LH level (4.6 mIU/mL; 1.4–15.0 mIU/mL in the follicular phase). She was referred to our hospital for further evaluation and treatment of hyperandrogenemia. Physical examination revealed that the patient was obese, with a body mass index of 30.4 kg/m^2^ (height, 160 cm; weight, 78 kg). Her arterial blood pressure was 150/89 mmHg, and her pulse was 72 beats/min. Notable features encompassed male pattern baldness, beard growth, and facial acne.

To rule out low LH levels due to possible hypopituitarism, hormonal blood tests and pituitary magnetic resonance imaging (MRI) were performed. The results ruled out the possibility of impaired LH elevation due to pituitary dysfunction. Her hormonal blood test results were as follows: LH, 1.9 mIU/mL; FSH, 3.8 mIU/mL; estradiol, 50 pg/mL; free testosterone, 7.78 ng/mL; prolactin, 13.6 ng/mL; ACTH, 32.4 pg/mL; cortisol, 9.4 µg/dL; and DHEAS, 195 µg/dL, with no significant abnormalities noted other than the elevated free testosterone level (Table 1). Pituitary MRI revealed no significant intracranial abnormalities, including the hypothalamus.

**Table 1:** Endocrine laboratory findings at initial presentation.

Parameter (unit)	Value
TSH (μIU/mL)	1.29
Free T4 (ng/dL)	1.15
LH (mIU/mL)	1.9
FSH (mIU/mL)	3.8
E2 (pg/mL)	50
Free testosterone (pg/mL)	7.78
PRL (mg/mL)	13.6
ACTH (pg/mL)	32.4
Cortisol (ug/dL)	9.4
DHEAS (ug/dL)	195

Abbreviations: ACTH, adrenocorticotropic hormone; DHEAS, dehydroepiandrosterone sulfate; E2, estradiol; free T4, free thyroxine; FSH, follicle‐stimulating hormone; LH, luteinizing hormone; PRL, prolactin; TSH, thyroid‐stimulating hormone.

Transvaginal ultrasonography at the referring clinic revealed a cystic lesion in the left ovary. Subsequent abdominal CT at our hospital demonstrated an 8 × 10 mm nodule‐like area in the left adrenal gland. A cystic lesion, suspected to be of ovarian origin, was also observed on the left side of the pelvis. Furthermore, CT revealed a seemingly normal right ovary; however, an area of enhancement on the ventral side was noted, suggesting a tumor (Figure 1). Subsequent pelvic MRI revealed a cyst of approximately 65 mm in diameter in the left half of the pelvis that appeared to be a left ovarian cyst. The right ovary appeared normal; however, the enhancing internal nodular‐like changes suggested a solid tumor (Figure 2).

Figure 1Contrast‐enhanced abdominal computed tomography (CT) images. (a) CT image revealing a nodule‐like area on the left adrenal gland (indicated by arrows), (b) CT image displaying a cystic lesion, likely of ovarian origin (indicated by arrows), and (c) CT image showing an enhancement area on the front side of the right ovary (indicated by arrows).

**Figure:**
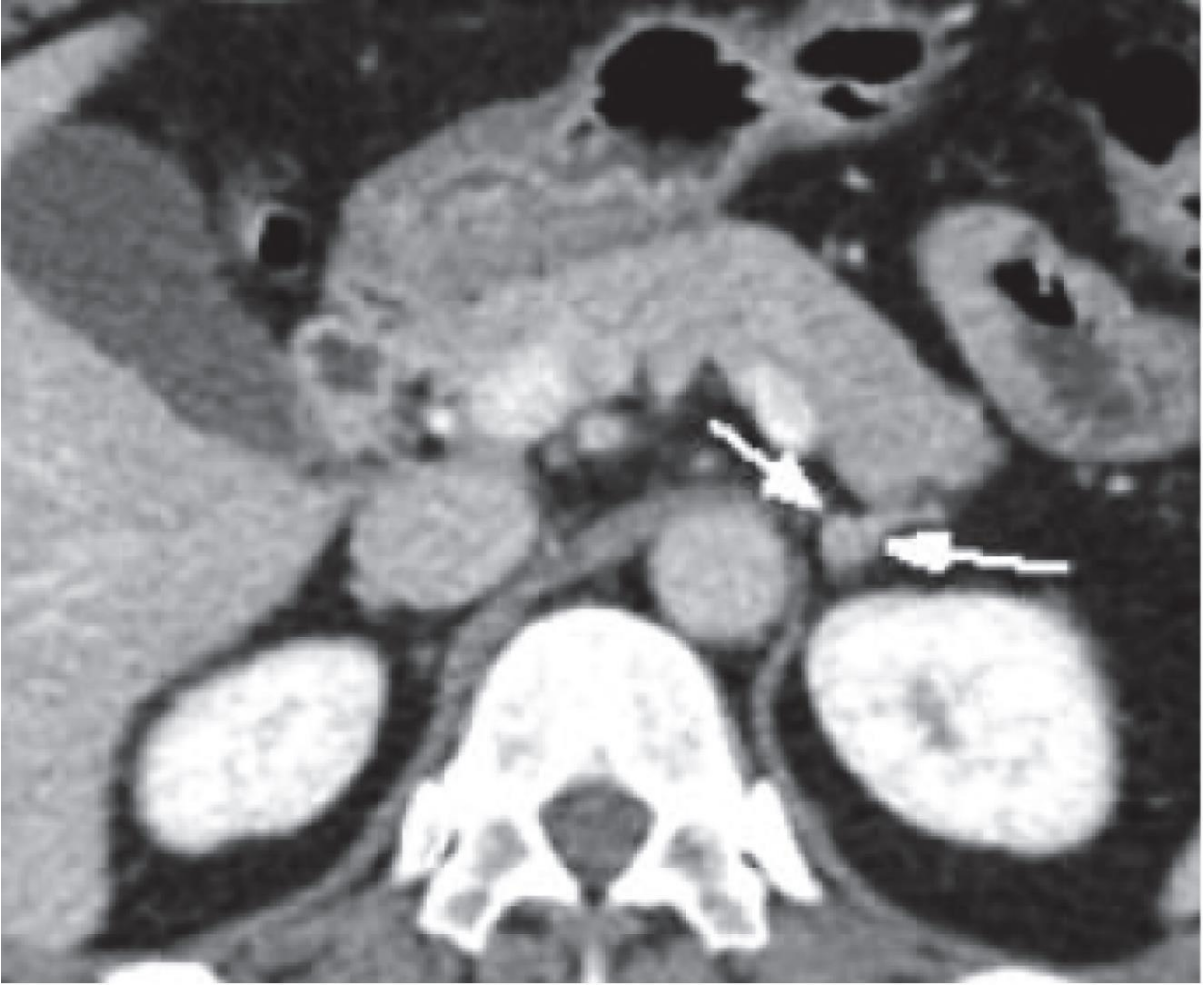
(a)

**Figure:**
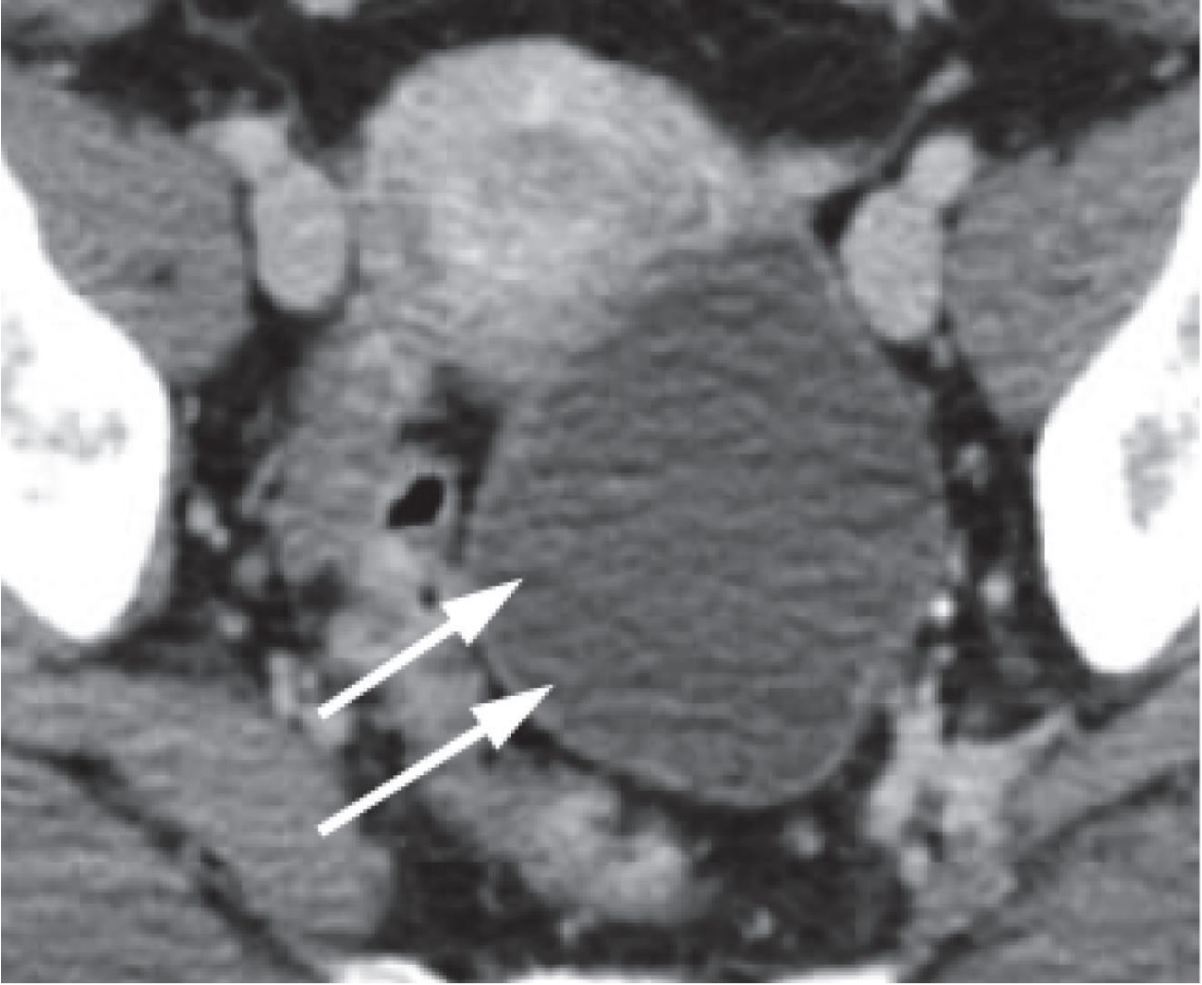
(b)

**Figure:**
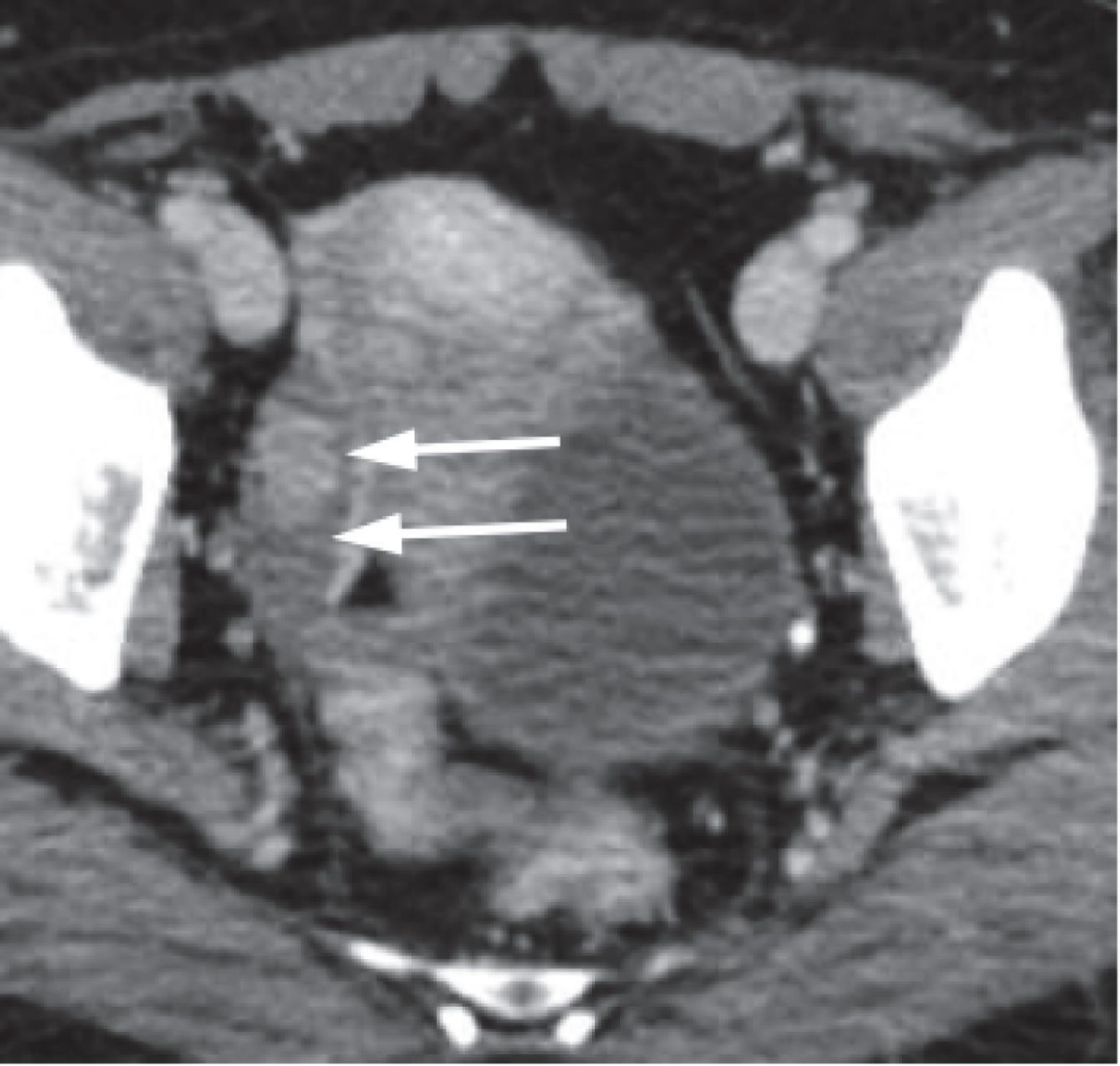
(c)

Figure 2Pelvic magnetic resonance images. (a) T2‐weighted image revealing a cyst ~65 mm in diameter on the left side of the pelvis, likely a left ovarian cyst (indicated by arrows) and (b) contrast‐enhanced T1‐weighted image with fat suppression demonstrating the right ovary with enhanced internal nodular‐like changes (indicated by arrows).

**Figure:**
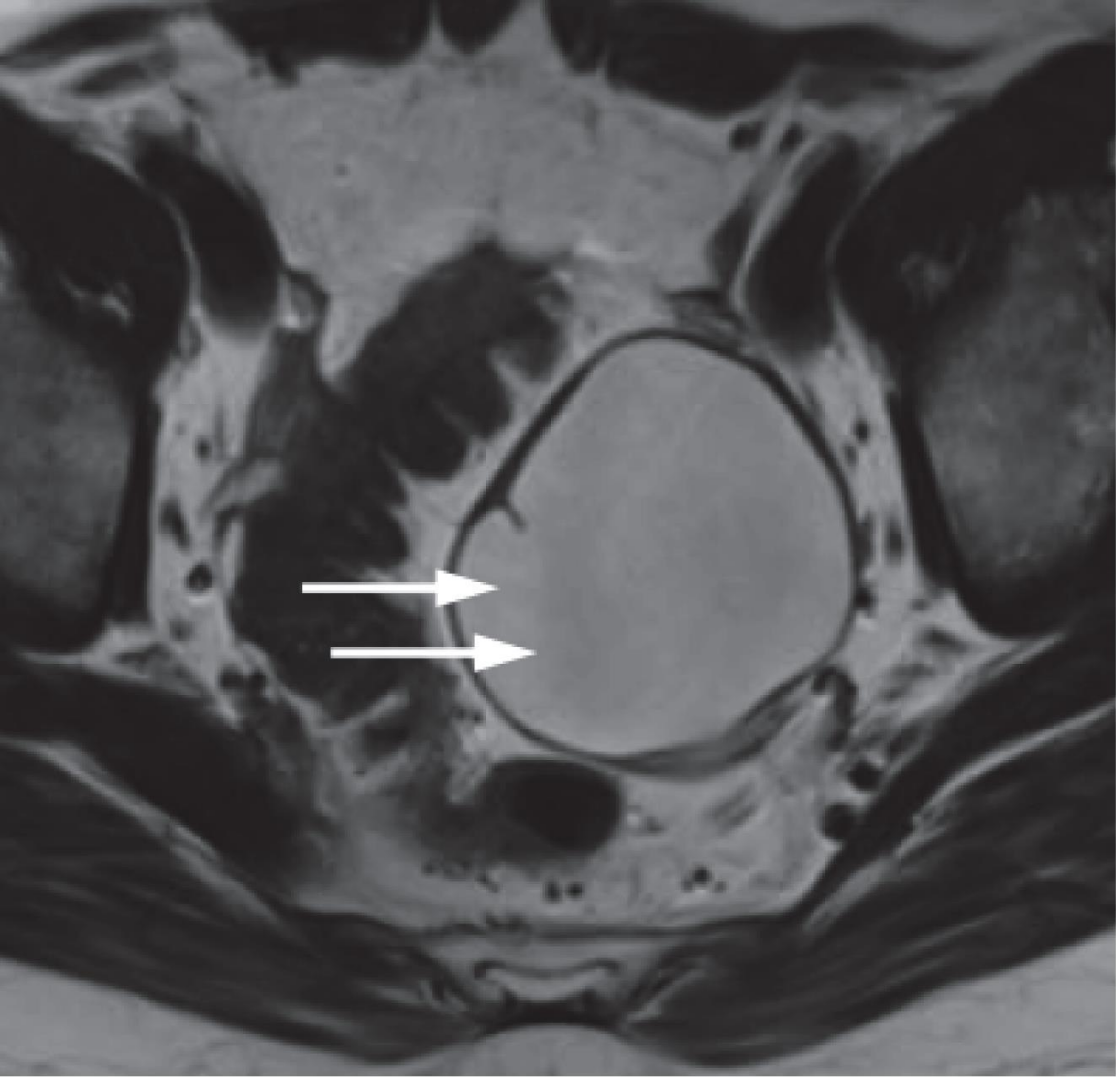
(a)

**Figure:**
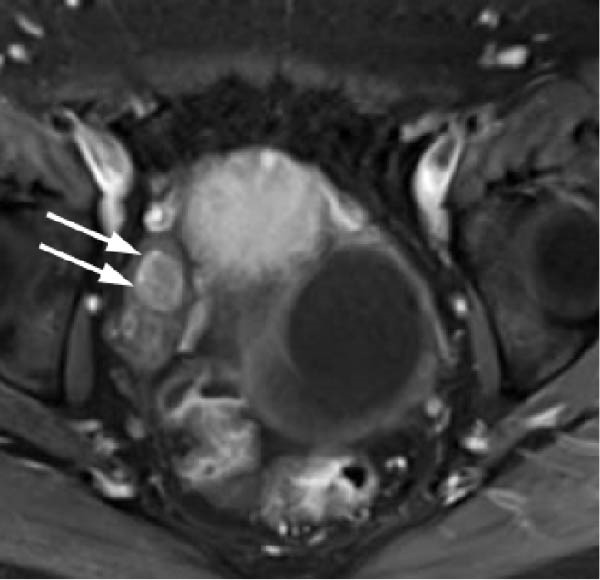
(b)

An LH–RH loading test was performed, which showed that the pre values and peak values of LH were 1.03 and 8.99 mIU/mL, respectively, whereas those of FSH were 2.85 and 9.39 mIU/mL, respectively. These results showed no increase in the LH level, ruling out the possibility of PCOS. Regarding the possibility of adrenal nodules, hormonal blood tests revealed no ACTH suppression, whereas late‐night blood and 1 mg dexamethasone suppression tests demonstrated cortisol suppression at 0.75 and 0.37 µg/dL, respectively. Moreover, no urinary cortisol level elevation was detected in the additional 24 h urine tests (27 and 28.8 µg/day) (Table 2). These findings suggested that the coexistence of ACTH‐independent Cushing’s syndrome was unlikely. Moreover, no increase in DHEAS level was noted, excluding the possibility of an increased adrenal‐derived testosterone level.

**Table 2:** Hormonal evaluation demonstrating elevated testosterone, suppressed gonadotropin, and normal adrenal steroid levels.

(A) LH–RH loading test						
Time (min)	0	15	30	60	90	120
LH (mIU/mL)	1.03	2.42	4.52	6.63	7.40	8.99
FSH (mIU/mL)	2.85	3.45	4.95	6.29	7.90	9.39
E2 (pg/mL)	48.3	—	—	—	—	50.1
Testosterone (ng/mL)	7.01	—	—	—	—	7.58

**(B) Late-night blood test**						

ACTH (pg/mL)	5.2					
Cortisol (µg/dL)	0.75					

**(C) 1-mg dexamethasone suppression test**						

ACTH (pg/mL)	≤1.5					
Cortisol (µg/dL)	0.37					

**(D) 24-hour urinary free cortisol excretion**	**Day 1**			**Day 2**		

Cortisol (µg/day)	27.0			28.8		

Abbreviations: ACTH, adrenocorticotropic hormone; E2, estradiol; FSH, follicle‐stimulating hormone; LH, luteinizing hormone.

Consequently, the patient was referred to a gynecologist under the presumption of excessive testosterone secretion of ovarian origin. Gynecological examination revealed clitoromegaly. Following consultation and a departmental case conference, a right ovarian solid tumor was suspected to be the source of hyperandrogenism. However, considering the patient’s desire for fertility preservation and her preference for treatment covered by national health insurance, a fertility‐sparing approach was selected. Furthermore, left ovarian lesions were present, and the likelihood of bilateral testosterone secretion could not be excluded. Laparoscopic partial resection was technically challenging owing to the small and fragile right ovarian tumor; excessive forceps manipulation risked crushing or rupturing the mass. Moreover, complete removal of the right ovary was considered the safest and most definitive treatment, as the likelihood of malignancy could not be completely ruled out. Therefore, OVS was planned to determine the laterality of androgen secretion and avoid unnecessary bilateral oophorectomy, and a radiologist was consulted to perform the procedure. As the right ovarian vein is generally challenging to visualize during sampling procedures [8, 12], preprocedural CT angiography was performed to better delineate the vascular anatomy (Figure 3). CT angiography delineated the courses and drainage patterns of the bilateral ovarian veins. The left ovarian vein drained into the caudal aspect of the left renal vein, 33 mm proximal to its confluence with the inferior vena cava (IVC). The right ovarian vein had two distinct drainage sites: one branch entered the IVC ventrally and caudally at the confluence with the right renal vein, located at the level of the transverse process of the first lumbar vertebra (L1), and the other branch drained into the anterior aspect of the IVC at the level of the inferior border of the second lumbar vertebra (L2).

Figure 3CT angiographic images illustrating the courses and drainage patterns of the bilateral ovarian veins. (a) Frontal view and (b) caudal view. The left ovarian vein drains into the left renal vein, 33 mm proximal to its confluence with the inferior vena cava (indicated by long arrows). The right ovarian vein has two drainage sites: one at the confluence with the right renal vein at the first lumbar (L1) level and another at the anterior aspect of the inferior vena cava at the L2 level (indicated by short arrows).

**Figure:**
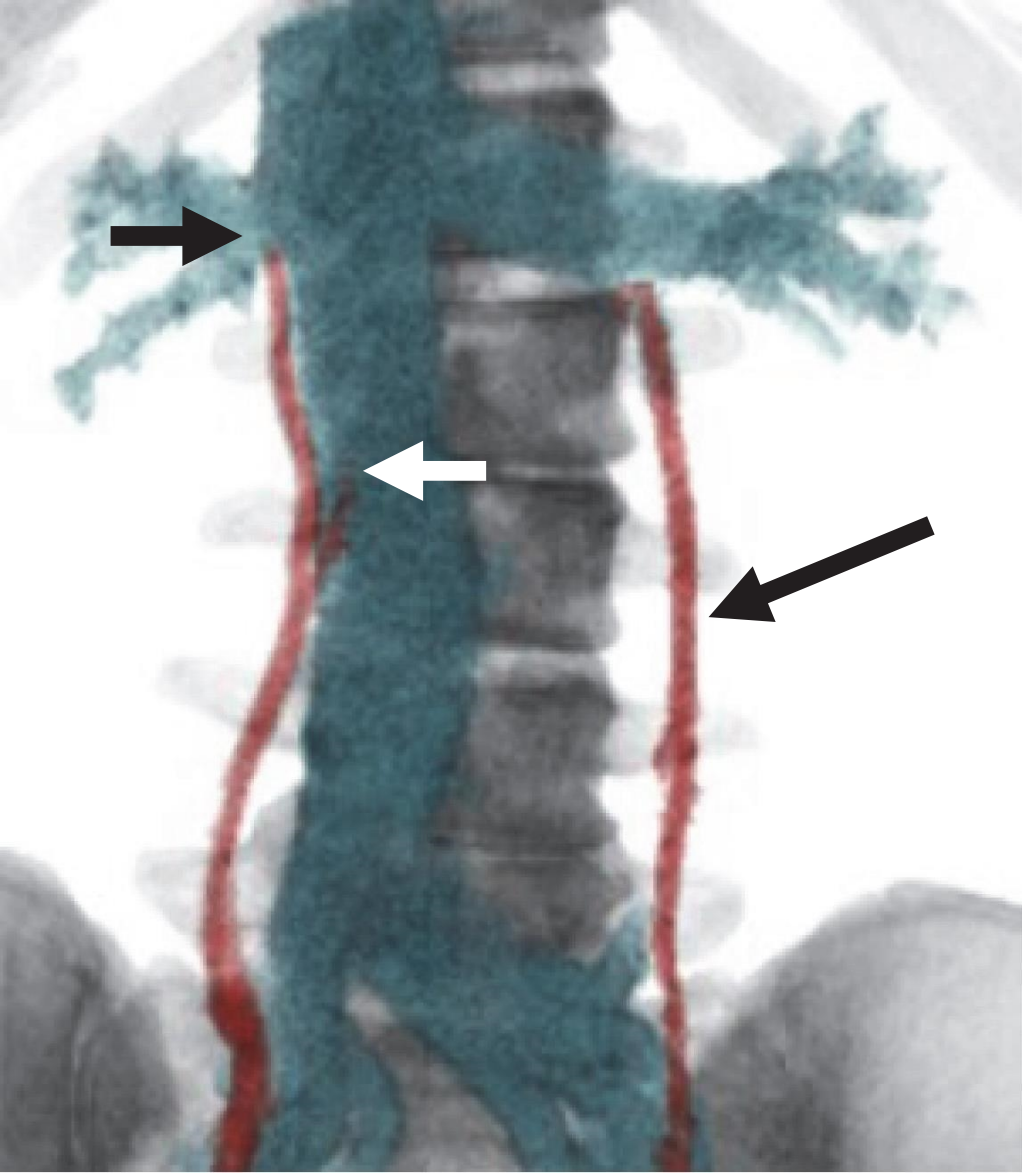
(a)

**Figure:**
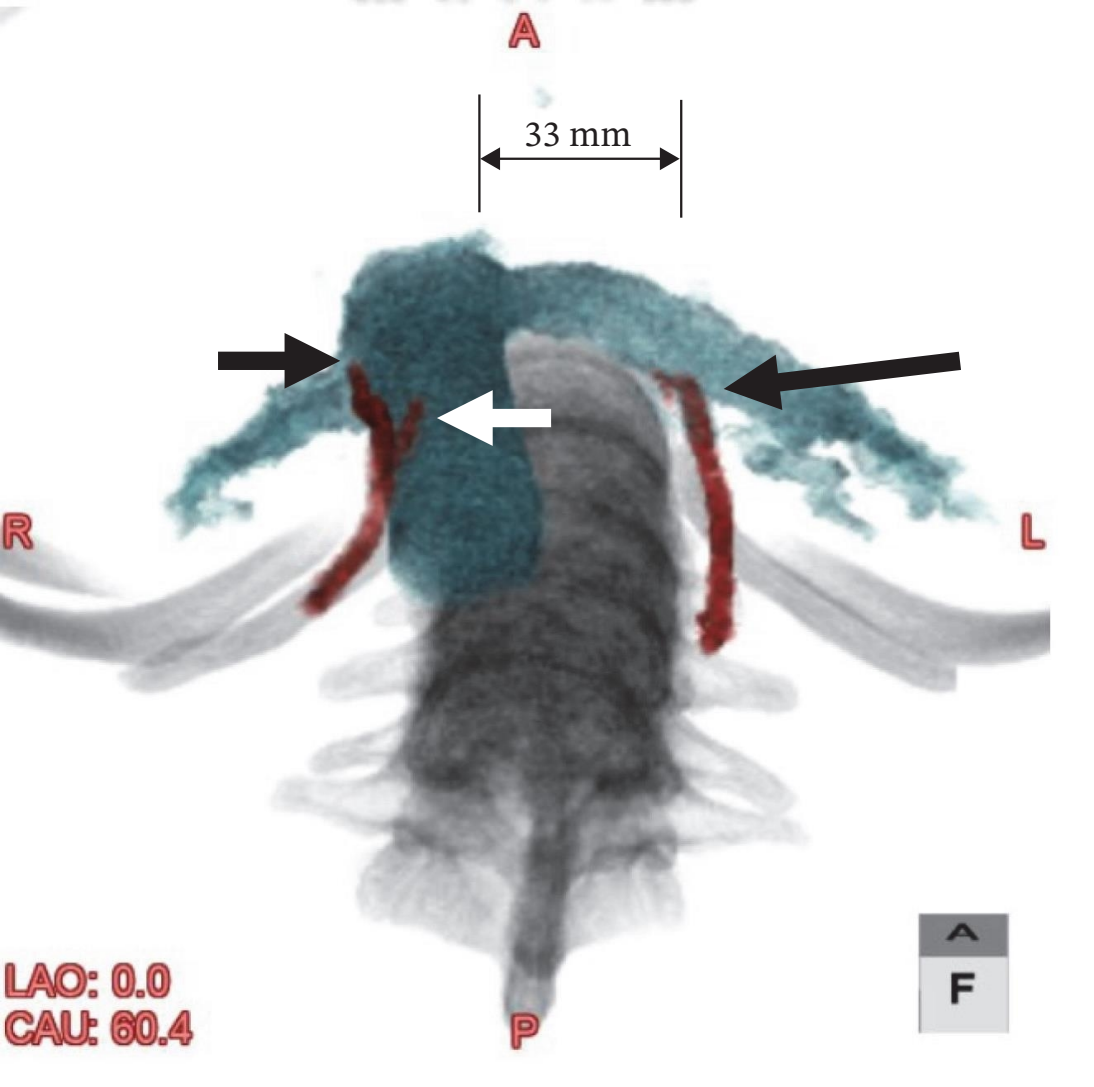
(b)

**Figure 4:**
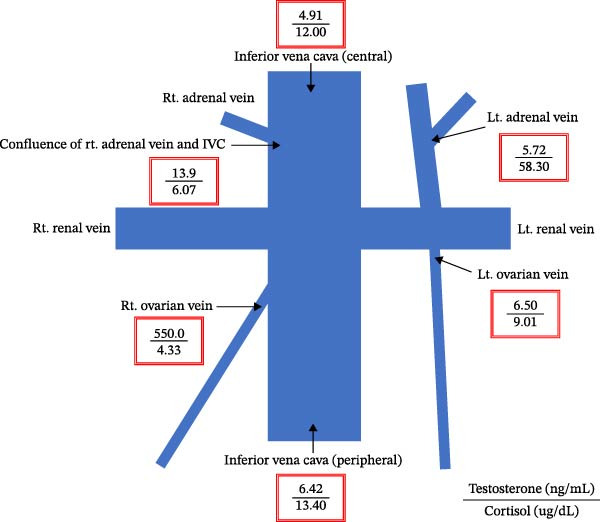
Ovarian vein sampling results.

These angiographic findings indicated that OVS was successfully conducted. Blood samples were collected from the IVC, bilateral ovarian veins, and bilateral adrenal veins for testosterone measurement (Figure 4). Serum testosterone levels were 4.91 and 6.42 ng/mL in the central and peripheral IVC, 13.9 and 5.72 ng/mL in the right and left adrenal veins, and 550.0 and 6.50 ng/mL in the right and left ovarian veins, respectively. Owing to difficulty in directly cannulating the right adrenal vein, sampling was performed near its confluence with the IVC. Cortisol levels measured in the right and left adrenal veins were 6.07 and 58.30 µg/dL, respectively, suggesting contamination of the right adrenal sampling site by ovarian venous return and explaining the elevated testosterone level.

**Figure 5:**
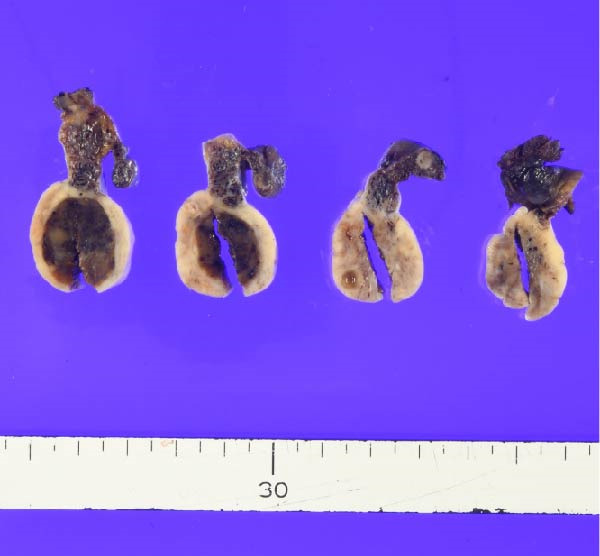
Ovarian tissue showing a solid brown tumor.

These findings indicated that excessive testosterone secretion was localized to the right ovary. A gynecologist subsequently performed laparoscopic right adnexectomy and left ovarian cystectomy. The right ovary was enlarged, and a 2.0 × 1.6 cm brownish substantial mass was observed within the ovary (Figure 5). The left ovarian cyst showed a 5.0 cm unilocular cyst, without a substantial component. At 2 days postoperatively, the patient’s blood testosterone level was measured and normalized to 0.17 ng/mL. Regarding pathological findings, macroscopically, the tumor was located at the ovarian hilum, and microscopically, it showed eosinophilic cells in the medullary area. No evidence of necrosis, hemorrhage, or fibrinoid degeneration of blood vessels was noted. Reinke’s crystals were not evident. Mitotic figures were absent. The degree of nuclear atypia was minimal. Immunohistochemical staining revealed positive results for calretinin and inhibin (Figure 6) and negative results for AE1/AE3, HMB45, Melan A, and SMA. Therefore, the final diagnosis was an ovarian Leydig cell tumor. Moreover, the Ki‐67 expression was approximately 1%. The left ovarian cyst was pathologically diagnosed as mucionous cystadenoma. The patient’s postoperative course was uneventful, resulting in her discharge from the gynecology department.

Figure 6(a) The cytoplasm is abundant and eosinophilic (hematoxylin and eosin, 40×), (b) tumor cells exhibiting diffuse cytoplasmic immunoreactivity for calretinin (40×), and (c) tumor cells exhibiting diffuse cytoplasmic immunoreactivity for inhibin (40×).

**Figure:**
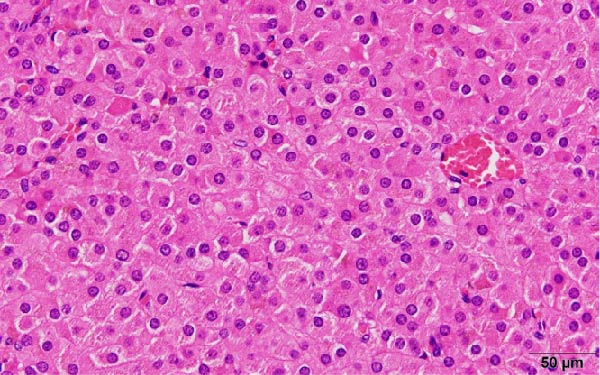
(a)

**Figure:**
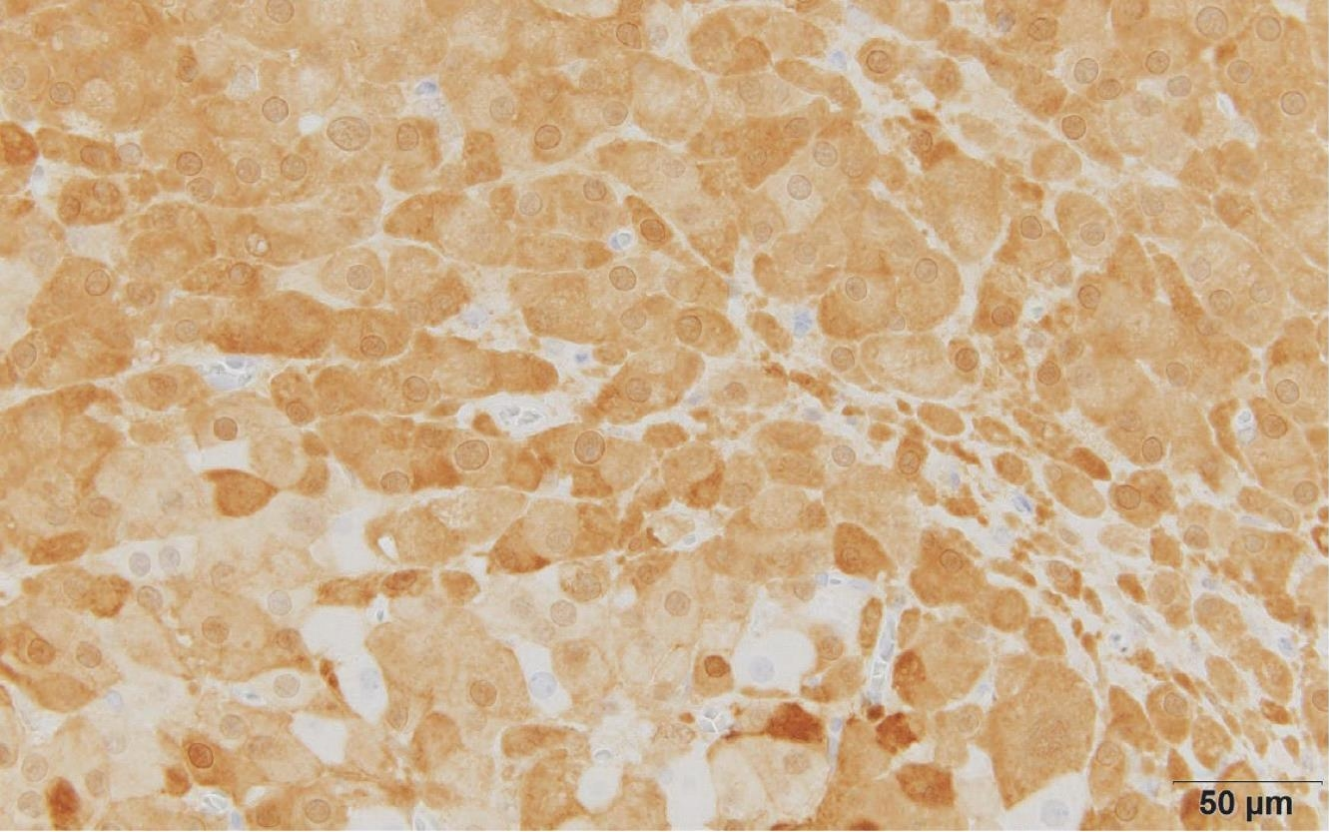
(b)

**Figure:**
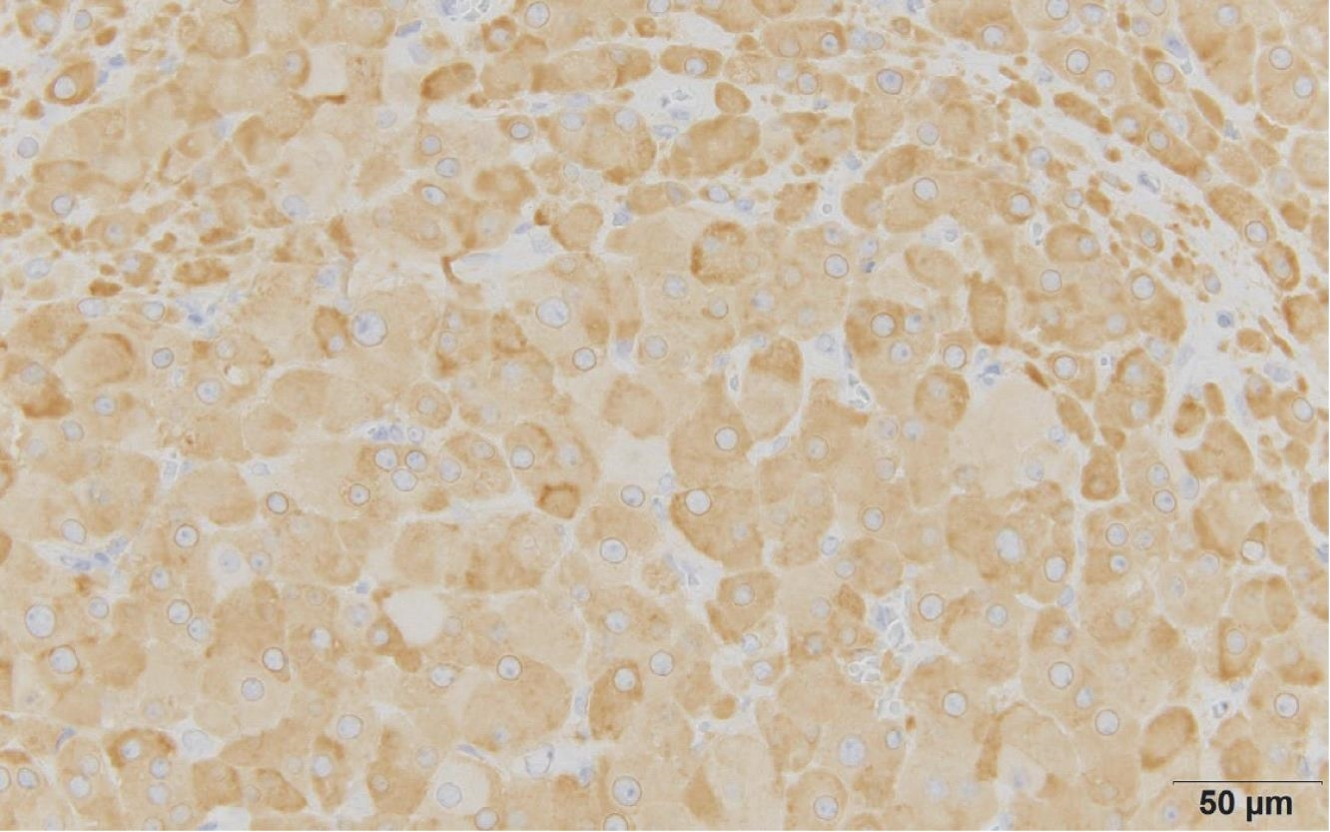
(c)

At 1 month postoperatively, her deepened voice had improved, and the frequency of hair removal treatments for her beard and anterior thighs had decreased. Gynecological examination also revealed improvement in clitoromegaly. Furthermore, her menstruation resumed. Despite the patient not noticing any improvement in her scalp hair loss, a dermatologist observed fine black hair regrowth, indicating early recovery. Subsequently, hypertrichosis and scalp hair loss gradually improved. At 3 months postoperatively, facial acne improved, and scalp hair continued to recover. Serum testosterone levels remained stable since surgery, and virilization symptoms gradually resolved within 1 year postoperatively. Regular follow‐up has been maintained to monitor for recurrence or hormonal imbalance, with no abnormalities observed to date. The overall clinical course is summarized in Table 3.

**Table 3:** Clinical timeline of symptom progression, diagnostic evaluation, interventions, and outcomes.

Time (months)	Clinical events and interventions	Findings/outcome
‐12	Onset of hirsutism and acne	Progressive symptoms
‐6	Voice deepening, clitoromegaly	Severe virilization
0	Presentation to hospital (Age: 42 years)	Testosterone 7.5 ng/mL; LH/FSH suppressed; DHEA‐S normal
0	Imaging (US, MRI, and CT)	No definite mass detected
+1	OVS with CT angiography	Right ovarian vein testosterone markedly elevated
+1	Laparoscopic right adnexectomy + left ovarian cystectomy	Right ovarian Leydig cell tumor (1.5 cm); benign left cyst
+0.5 – + 2	Postoperative follow‐up	Testosterone normalized; menses resumed
+12	Long‐term follow‐up	Resolution of virilization
+18	Ongoing follow‐up	Regular cycles, no recurrence

## 3. Discussion

Ovarian Leydig cell tumors, extremely rare androgen‐producing neoplasms, typically cause rapidly progressive virilization, such as hirsutism and voice deepening, and may also lead to secondary amenorrhea. Although significantly elevated serum testosterone levels raise suspicion for an androgen‐secreting tumor, preoperative localization remains a major challenge, particularly in cases with bilateral ovarian abnormalities or small lesions that are not readily detected using conventional imaging modalities. Previous reports have indicated unnecessary bilateral oophorectomy when imaging failed to identify a unilateral lesion, highlighting the clinical significance of functional localization techniques [6–8].

In this case, no discrete lesion was identified despite the use of advanced imaging modalities, including CT and MRI (Figures 1 and 2). However, the patient’s rapid clinical progression and severe biochemical hyperandrogenism prompted further evaluation. The clinical timeline (Table 3) illustrates the discordance between progressive symptoms and negative imaging, emphasizing the significance of functional testing.

When imaging is inconclusive, OVS has been suggested as a definitive method for localizing androgen excess. Its utility is particularly evident in radiologically occult tumors, where it facilitates accurate lateralization and guides targeted surgery [6, 13, 14]. Nevertheless, OVS is technically demanding, particularly for the right ovarian vein owing to its drainage pattern into the IVC [8, 12]. In our patient, preprocedural CT angiography (Figure 3) facilitated successful catheterization, and selective sampling confirmed unilateral androgen secretion (Figure 4). This diagnostic pathway underscores how advances in AVS methodology can be adapted to OVS to improve success rates and clinical applicability [10, 15, 16].

These findings directly influenced surgical decision‐making. A fertility‐sparing approach, right adnexectomy with contralateral cystectomy, was possible considering the functional confirmation of unilateral androgen excess. Gross pathology demonstrated a 2.0 × 1.6 cm Leydig cell tumor (Figure 5), and histopathological examination confirmed the diagnosis (Figure 6). The clinical outcomes, including biochemical normalization, menstruation resumption, and virilization regression, further support the effectiveness of this strategy (Table 3).

This case emphasizes three key lessons. First, in reproductive‐aged females with severe hyperandrogenism and inconclusive imaging, particularly those desiring fertility preservation, SVS should be promptly considered to prevent unnecessary bilateral surgery [6, 7, 11–14, 17]. Second, adjunctive CT angiography can overcome the technical limitations of OVS, reflecting broader innovations in endocrine interventional radiology [4, 5, 10, 12, 15, 16]. Lastly, accurate localization directly impacts surgical planning, enabling FSS and reproductive potential preservation in young females [18, 19].

Furthermore, the successful application of CT angiography‐assisted OVS in a reproductive‐aged female with bilateral ovarian abnormalities and severe hyperandrogenism represents the distinctive feature of this case. Most previous reports of OVS have encompassed postmenopausal females, whereas this case underscores its clinical utility in a younger patient desiring fertility preservation, demonstrating that this combined technique can achieve accurate lateralization and facilitate FSS even when conventional imaging is inconclusive.

Collectively, this case demonstrates the evolving role of functional endocrine diagnostics in rare ovarian tumors. Clinicians can refine localization strategies, optimize surgical decision‐making, and ultimately enhance oncologic and reproductive outcomes by integrating the established AVS methodology into OVS.

## 4. Conclusion

This case demonstrates that CT angiography‐assisted OVS can successfully localize androgen‐producing ovarian tumors when conventional imaging is inconclusive, facilitating FSS in reproductive‐aged females. Integrating the AVS methodology into OVS may represent a valuable diagnostic approach in selected cases of severe hyperandrogenism.

## Funding

No funding was received for this study.

## Ethics Statement

Written informed consent was obtained from the patient for publication of this case report and any accompanying images. Ethics approval was waived according to institutional policy for single‐patient case reports.

## Consent

The patient gave informed consent for the publication of this case report. However, the patient did not authorize the use of photographs of the face or perineum.

## Conflicts of Interest

The authors declare no conflicts of interest.

## Patient Perspective

The patient was relieved to receive an accurate diagnosis and was satisfied with the fertility‐sparing surgery. She stated that the improvement in hyperandrogenic symptoms and the preservation of reproductive potential were particularly significant outcomes.

## Data Availability

All data generated or analyzed during this study are included in this published article.
